# Ovarian Cancer Ascites Enriched for CCL23 Reduces Macrophage-Derived CXCL10 Secretion and Is Associated with Poor Patient Outcomes

**DOI:** 10.3390/cancers17243925

**Published:** 2025-12-08

**Authors:** Susan M. Lang, Supreeti Tallapragada, Justine Chan, Oliver Dorigo

**Affiliations:** 1 Department of Obstetrics & Gynecology, Division of Gynecologic Oncology, Stony Brook University, Stony Brook, NY 11794, USA; 2 Department of Obstetrics and Gynecology, Division of Gynecologic Oncology, Stanford School of Medicine, Stanford Cancer Institute, Stanford University, Palo Alto, CA 94304, USA

**Keywords:** ovarian cancer, tumor immune microenvironment, cytokine profiling, prognostic biomarker, CCL23, ascites

## Abstract

**Background**: Ovarian cancer ascites contributes to the immunosuppressive tumor microenvironment (TME) via macrophage-derived chemokine ligand 23 (CCL23) signaling, T-cell exhaustion, and upregulating pro-inflammatory cytokines. However, the extent to which ascites-derived CCL23 concentrations associate with changes in pro- and anti-inflammatory cytokines and overall patient survival in ovarian cancer patients remains unknown. **Methods**: CCL23 concentrations and pro-inflammatory cytokines were measured from ascites of stage III and IV epithelial ovarian cancer patients by ELISA and Luminex assays, respectively. Kaplan–Meier survival analysis was performed using patient outcome data from Stanford University Hospital and the Cancer Genome Atlas. The impact of CCL23 peptides on pro-inflammatory cytokine secretion was evaluated in vitro using differentiated THP-1 monocytes. **Results**: A total of 40 patients were enrolled and CCL23 concentrations were detected in all ascites samples (median = 2.42 ng/mL; range [0.06–6.45]). Reduced survival time corresponded with high CCL23 containing samples (mOS: 3.2 years, [3.9 ng/mL]) versus intermediate (mOS: 6.0 years, [2.5 ng/mL]) or low (mOS: 5.9 years; [1.4 ng/mL]) groups. TGCA analysis of patient outcomes was confirmatory. A significant negative correlation was observed between high CCL23 ascites concentrations versus CXCL10 and soluble PD-1 cytokine levels. High tumor expression of CXCL10 was associated with improved survival (mOS; 5.9 years) versus low CXCL10 expression (mOS; 3.2 years). In vitro, CCL23-stimulated THP-1 macrophages exhibited reduced CXCL10 secretion via STAT-3 activation. **Conclusions**: High CCL23 concentrations in ovarian cancer ascites reduces CXCL10 secretion from myeloid cells and associates with reduced patient survival.

## Simple Summary

Advanced ovarian cancer is often associated with a fluid called ascites that supports tumor growth and suppresses the body’s immune response. This study explores how a specific immune signal, CCL23, found in the ascites of ovarian cancer patients, affects the tumor microenvironment and patient survival. By measuring CCL23 and related immune molecules in patient samples and testing their effects in laboratory models, it was found that higher levels of CCL23 were linked to reduced survival and lower levels of a protective immune signal called CXCL10. These findings suggest that CCL23 may help tumors evade immune defenses by suppressing beneficial immune activity. Understanding this process may lead to new strategies for improving immune-based treatments for ovarian cancer.

## 1. Introduction

Ovarian cancer is a leading cause of female cancer-related mortality in the United States and remains difficult to treat due to the increased frequency of late-stage diagnosis and platinum resistant recurrence [1,2]. Although novel therapies such as immune checkpoint inhibitors are effective in preventing tumor metastasis and improving patient survival when used in other gynecologic malignancies, these same therapies underperform in ovarian cancer clinical trials [3,4]. One hypothesis for the reduced clinical performance of immune checkpoint inhibitors in ovarian cancers is the presence of a highly immunosuppressive tumor microenvironment (TME) [4,5].

The formation of the TME is supported by the presence of malignant ascites. Ascites fluid acts as a reservoir of pro-inflammatory and immunosuppressive cytokines that promotes advancement or recurrence of disease via reduced immune cell function [6]. As such, the presence of ascites on preoperative imaging has been independently associated with increased postoperative morbidity at the time of debulking surgery, as well as a lower likelihood of achieving complete gross resection [7]. Moreover, the presence of ascites is associated with worsening oncologic outcomes, possibly reflecting a more aggressive biological behavior of ovarian cancers [7,8]. In patients with high grade serous ovarian cancer presenting with ascites, low-volume ascites exhibited distinct molecular features whereas those with high-volume ascites were characterized by the beneficial upregulation of immune-related genes and tumor-infiltrating cells [9]. Thus, the unique mixture of ascites-derived cytokines, as well as the activation of their cognate receptors within the TME, drives cancer cell proliferation, immune cell modulation, and metastasis in ovarian cancers [10,11].

One pro-tumorigenic cytokine that contributes to the TME is CC chemokine ligand 23 (CCL23). CCL23 is a potent chemoattractant for myeloid cells that stimulates chemotactic migration of endothelial cells to support tumor angiogenesis in ovarian tumors and increases the proportion of exhausted CD8+ T-cells in the TME [12,13,14]. For example, previous studies in our laboratory highlight the role of macrophage-secreted CCL23 promotes the migration and colonization of ovarian cancer cells to the omentum via the CCR1 signaling axis [15]. Notably, genetic deletion or pharmacologic inhibition of the murine-homolog of CCR1 reduces CCL23-induced immune cell infiltration into the omentum [12,15]. Prior work from our laboratory has also found that CCL23 signaling is necessary and sufficient to promote ovarian cancer cell migration as well as metastasis from solid ovarian cancer tumors in the homologous murine pathway [15]. Despite these established roles in metastasis and immune exhaustion, the specific contribution of ascites-derived CCL23 to immune modulation in human ovarian cancer has not been characterized. In particular, whether CCL23 levels in human ascites correlate with patient outcomes or influence key immunomodulatory cytokines remains unknown.

In the present study, we aimed to address these gaps by integrating primary human ascites cytokine profiling through patient survival analyses from both institutional and TCGA datasets and in vitro mechanistic experiments. We hypothesize that human ovarian cancer ascites act as a reservoir of CCL23, and that high CCL23 concentrations promote an immunosuppressive phenotype and adverse patient outcomes. We measured ascites-derived CCL23 concentrations and changes in pro-inflammatory cytokines from ovarian cancer patients’ samples and further evaluated survival from patient outcome data. Using an in vitro culture model, we assessed the influence of CCL23 stimulation on secreted cytokines that were identified as differentially expressed in patient samples.

## 2. Materials and Methods

**Patient samples.** All specimens were obtained after informed patient consent and according to the approved protocol of the Institutional Review Board (IRB) #42966 at Stanford University Hospital. Ascites samples were collected from patients with newly diagnosed and recurrent ovarian cancer and stored at Stanford University. Analysis was performed on a total of 40 patient samples with corresponding clinical data.

**Enzyme-linked immunoassay (ELISA).** The concentrations of CCL23 (Cat# EHCCL23; Thermo Scientific, Waltham, MA, USA) and CXCL10 (Cat# KAC2362; Thermo Scientific, Waltham, MA, USA) were determined by ELISA according to the manufacturer’s protocol. Briefly, ascites samples stored at −80 °C were thawed, stored on ice, and centrifuged (1400 RPM for 10 min at 4 °C). Samples were diluted 1:2 (CCL23) and 1:10-20 (CXCL10). Samples were run in triplicate.

**Multiplex assay system for the quantitation of human cytokines.** The Human Immune Monitoring Center (HIMC) at Stanford University performed custom Luminex assays (EMD Millipore Corporation, Burlington, MA, USA) on cell-free ascites samples prepared from raw ascites using centrifugation (1400 RPM for 10 min at 4 °C) and supernatant harvest. Three cytokine panels were measured using H80 kits (EMD Millipore Corporation, Burlington, MA, USA) including the following: panel 1 is Milliplex HCYTA-60K-PX48 (Merck, Darmstadt, Hesse, Germany); panel 2 is Milliplex HCP2MAG-62K-PX23 (Merck, Darmstadt, Hesse, Germany); and panel 3 includes the Milliplex HSP1MAG-63K-06 and HADCYMAG-61K-03 (Resistin, Leptin and HGF) (Merck, Darmstadt, Hesse, Germany) to generate a 9 plex. The panels were run according to the manufacturer’s recommendations through the HIMC. Briefly, samples were diluted 3-fold (panels 1 and 2) or 10-fold for panel 3. Samples were run in duplicate. Diluted samples (25 µL) were mixed with antibody-linked magnetic beads in a 96-well plate and incubated overnight at 4 °C with orbital shaking (500–600 RPM). Samples were washed twice using BioTek ELx405 buffer (BioTek Instruments, Winooski, VT, USA) and incubated at room temperature with biotinylated detection antibody for 1 h. Streptavidin-PE was added for 30 min with shaking. Plates were washed as described above and phosphate-buffered saline (PBS) was added to wells for reading in the Luminex FlexMap3D Instrument (Austin, TX, USA) with a lower bound of 50 beads per sample per cytokine. Each sample was measured in duplicate. Custom Assay Chex beads (Radix BioSolutions, Georgetown, TX, USA) were added to all wells as a control. Bead counts <20 were excluded.

**Cell culture.** THP-1 human monocytic cell lines (ATCC #TIB-202, Manassas, VA, USA) were cultured in RPMI 1640 Medium (Corning Cellgro, Manassas, VA, USA; 10-041-CM) supplemented with 10% fetal bovine serum (FBS, Bio-Techne, Minneapolis, MN, USA; S1155OH) and 1% penicillin/streptomycin (Corning Cellgro, Manassas, VA, USA; Cat # 30-002-Cl). Media for THP-1 cells was additionally supplemented with 0.01% beta-mercapto-ethanol (Sigma-Aldrich, St. Louis, MO, USA; Cat #M3148-250ML). THP-1 monocytes were differentiated into M0 macrophages via application of phorbol 12-myristate-13-acetate (PMA) to a final concentration of 10 ng/mL for a 48 h differentiation period. The cells were grown at 37 °C in a 5% CO_2_ incubator. Cells were serum-starved using 0.5% serum for 6 h prior to experiments. Cell-free supernatant was harvested from serum-starved cells incubated with vehicle, CCL23 aa22-120 (Peprotech, East Windsor, NJ, USA; 300-29) or CCL23 aa46-120 (R&D Systems, Minneapolis, MN, USA; 131-M1-025) peptides for 30 min, or the signal transducer and activator of transcription (STAT-3) inhibitor NSC 74859 (100 μM; Tocris; Minneaoplis, MN, USA; 4655) was reconstituted and serum-starved cells were incubated with 100 μM NSC 74859 for 30 min [16] prior to incubation with CCL23. The 30 min time-point and concentration of 100 ng/mL for CCL23 was chosen based on data from published datasets for inducing cancer cell migration and metastasis [15].

The HEL 92.1.7 erythroblastic cell line (ATCC, Manassas, VA, USA) was cultured in RPMI supplemented with 10% FBS and 1% penicillin/streptomycin. Differentiation into M0 macrophages was completed as above.

**RNA Isolation, cDNA synthesis, and qRT-PCR.** Total RNA was extracted using the RNeasy Mini Plus Kit (Qiagen, Germantown, MD, USA; 74134) and was measured using NanoDrop (Thermo Scientific, Waltham, MA, USA). cDNA was generated using the Superscript Strand III cDNA SuperMix (Invitrogen; Waltham, MA, USA; 18-080-051) according to the manufacturer’s instructions. Real-time PCR was performed using the SSO Fast Evergreen SuperMix (Biorad; Hercules, CA, USA; 172-5211) on an Applied Biosystems 7500 Fast Real-Time PCR System (Applied Biosystems, Waltham, MA, USA). Expression of the target genes was normalized to the housekeeping gene 18S. RNA from M0 macrophages differentiated from the THP1 monocytic cell line was used as a control for these gene expression studies. RNA from M0 macrophages prior to stimulation were assessed for baseline CCR1 expression. Primers were obtained from ELIM Biopharm (Hayward, CA, USA; human CCR1 forward AGCTGTCCGTTTGATTTTTGTCA; human CCR1 reverse CCAGGTCCAAATGTCGCTCT). All real-time RT-PCRs were performed in triplicate and the relative mRNA expression of each target gene was determined by using the formula 2^−ΔCT^ (CT, cycle threshold) where ΔCT = CT (target gene) − CT (18S). The comparative expression level of each target gene between different samples was 2^−ΔΔCT^.

**Immunocytochemistry (ICC).** Coverslips were prepared with 0.1% poly-L-lysine (Sigma Aldrich, Burlington, MA, USA; P8920) in a 6-well cell culture plate (Corning, Glendale, AZ, USA; 3516) and then washed with 1× PBS (Corning Cellgro; 21-040-CV). Cells at 2 × 10^4^ were added to each well and differentiated to M0 macrophages as above. Cells were serum-starved for 6 h and then treated with vehicle (1× PBS), mature CCL23 aa22-120 (Peprotech, East Windsor, NJ, USA; 300-29), or truncated CCL23 aa46-120 (R&D Systems, Minneapolis, MN, USA; 131-M1-025) for 30 min. Media was aspirated and cells were fixed with 4% paraformaldehyde (PFA; Santa Cruz Biotechnology, Dallas, TX, USA; Cat# sc-281692) solution for 15 min. Coverslips were washed with 1× PBS. Coverslips were incubated with 0.1% Triton X-100 (Fisher Scientific, Hampton, NH, USA; BP151-100) in 1× PBS for 15 min. Coverslips were washed three times. Coverslips were incubated in a 1% BSA blocking solution for 1 h at room temperature. Slides were incubated with primary antibody overnight and subsequent secondary antibodies. Cells were incubated with 4′,6-diamidino-2-phenylindole (DAPI; Thermo Scientific; D1306) for 15 min. After washing, coverslips were mounted on slides using mounting media (VectaShield Mounting Media; Vector Laboratories, Burlingame, CA, USA; Cat# H-1000-10), allowed to dry, and then sealed. Slides were then imaged on an EVOS Fluorescence microscope (Thermo Fisher Scientific, Waltham, MA, USA). Level of intensity of cell staining was determined by ImageJ software (Version 2.3.0).

**Statistical Analysis.** All data were analyzed using GraphPad Prism v10.0 software. A Shapiro–Wilk test was performed to determine normality and a Brown–Forsythe/Bartlett’s tests were used to determine equal variance. Data that passed normality and equal variance tests were analyzed using Student’s *t*-test or ANOVA for three or more groups. Data that did not pass normality were log transformed for parametric testing. When significant F values were obtained, an independent or paired *t*-test with a layered Bonferroni correction was performed. If a dataset failed normality, a Kruskal–Wallis test with Dunn’s multiple comparison was performed. In all instances, the statistical conclusions were similar between the non-parametric and parametric tests. Data are presented as mean ± S.E.M. plus individual data points when possible, with statistical significance; * *p* < 0.05. Kaplan–Meier survival analysis was performed on patient data. Log-rank and Cox proportional hazards tests were performed to test for significance; * *p* < 0.05. The KM plotter online tool (http://kmplot.com) was used to evaluate the prognostic significance of CCL23 and CXCL10 expression in ovarian cancer. Survival analysis was conducted for stage III and IV high grade serous samples with an auto-select best cutoff feature. Log-rank tests were performed for significance; *p < 0.05. K-means clustering was performed in Microsoft Excel using the built-in Analysis ToolPak (version 16.103.3), after first standardizing (z-scaling) all variables to ensure equal weighting. Optimal cluster cutoffs were validated by comparing within-cluster sum of squares across repeated runs.

## 3. Results

### 3.1. Ovarian Cancer Ascites with High CCL23 Concentrations Associated with Poor Patient Outcomes

Ovarian cancer ascites samples were collected from newly diagnosed and recurrent ovarian cancer patients (N = 40) (Table 1).

**Figure 1:**
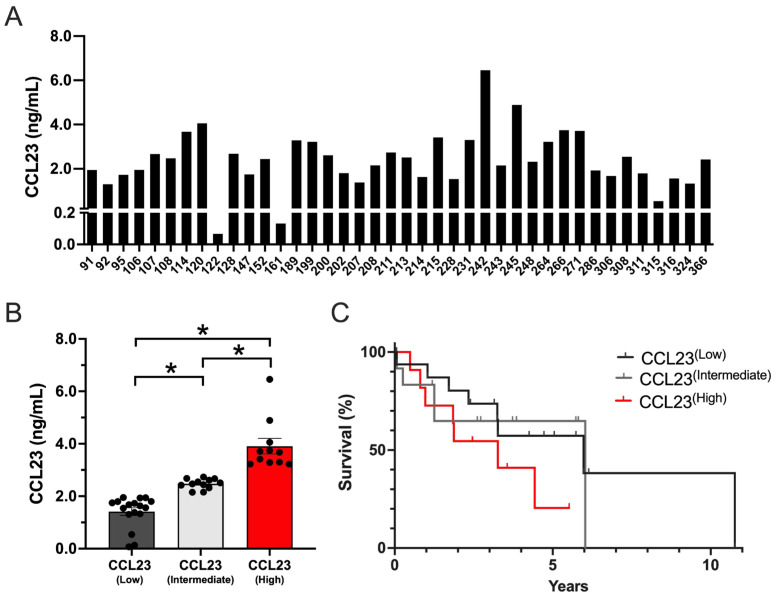
CCL23 is present in all human ascites samples with primary or recurrent ovarian cancer. (**A**) The distribution of CCL23 concentration (ng/mL) in ovarian cancer ascites samples (*n* = 40). (**B**) Low (*n* = 17), intermediate (*n* = 12), and high (*n* = 11) CCL23 ascites concentrations. Data presented as mean ± S.E.M. ANOVA with Bonferroni post hoc test performed for significance; * *p* < 0.05. (**C**) Kaplan–Meier survival curves (log-rank) of patient outcome data from low (*n* = 17; gray line), intermediate (*n* = 12; light gray line), and high (*n* = 11; red line) CCL23 concentrations.

CCL23 concentrations were measured using ELISA (Figure 1A). The median concentration was [2.42] ng/mL and ranged between [0.06 and 6.45] ng/mL.

No significant differences were detected between CCL23 concentrations based on different tumor or treatment characteristics such as histology, treatment exposure, or recurrence (Supplementary Figure S1). Using K-means cluster analysis, three distinct patient groups were identified in which CCL23 concentrations were significantly different (*p* ≤ 0.05), including low (1.41 ± 0.15 ng/mL; *n* = 17), intermediate (2.47 ± 0.06 ng/mL; *n* = 12), and high (3.91 ± 0.29 ng/mL; *n* = 11) groups (Figure 1B). Kaplan–Meier survival analysis was performed on patient outcome data. Patients with high CCL23 concentrations corresponded with shorter survival times versus low CCL23 concentrations (HR 1.83 [95% CI 0.66–5.05], log-rank *p* = 0.18) or intermediate CCL23 concentrations (HR 1.85 [95% CI 0.58–5.82], log-rank *p* = 0.29) (Figure 1C). Although these data were not significantly different in our model, they were clinically meaningful (mOS; 3.2 years for CCL23 high patients versus 5.9 years for CCL23 low patients and 6.03 years for CCL23 intermediate patients).

### 3.2. CXCL10 and Soluble PD-1 Concentrations Are Reduced in Ovarian Ascites Samples Containing High Concentrations of CCL23

**Figure 2:**
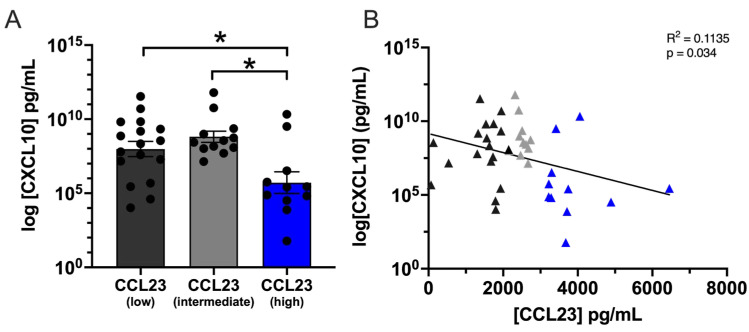
High CXCL10 levels correlate with low CCL23 concentrations in ovarian ascites. (**A**) Quantification of C-X-C Motif Chemokine Ligand 10 (CXCL10) cytokine concentration from ovarian ascites patient samples containing low (*n* = 17), intermediate (*n* = 12), and high (*n* = 11) CCL23. Data presented as mean ± S.E.M. Statistical analyses performed using one-way ANOVA with Dunnet’s post hoc test for significance; * *p* < 0.05. (**B**) Pearsons linear regression of CXCL10 versus CCL23 concentrations indicated a significant negative association between (F(1,38) = 4.86, *p* = 0.033).

CCL23-mediated signaling increases pro-inflammatory cytokine expression in the TME, yet it was unclear whether ovarian cancer ascites also harbored differences in other pro- and anti-inflammatory cytokines. We evaluated the composition of ovarian cancer ascites for 95 unique markers in patient samples containing high, intermediate, and low CCL23 levels. Among these 95 unique markers, only CXCL10 and soluble PD-1 were significantly different between patient samples. No significant differences were observed for the remaining markers (Supplemental Table S1). A significant difference was detected between groups for CXCL10, which is presented as a log transformed value (Figure 2A).

CXCL10 concentrations were significantly lower between CCL23 high (5.70 ± 0.73 pg/mL, *n* = 11) and CCL23 intermediate (8.81 ± 0.38 pg/mL, *n* = 12) or CCL23 low (7.99 ± 0.51 pg/mL, *n* = 17) samples (*p* < 0.05). A significant negative correlation was observed between the logCXCL10 and CCL23 concentrations (r(38) = −0.34, *p* < 0.05) (Figure 2B). Linear regression indicated a significant negative association between CXCL10 and CCL23 (F(1,38) = 4.863, *p* = 0.033). Similarly, PD-1 concentrations were significantly lower between CCL23 high (210 ± 38.12 pg/mL, *n* = 11) and CCL23 intermediate (429.6 ± 55.50 pg/mL, *n* = 12) or CCL23 low (468.3 ± 81.08 pg/mL, *n* = 17) samples (*p* < 0.05) (Figure S2). No significant group differences were observed between patient samples for all other measured cytokines (Supplemental Table S1).

### 3.3. Ovarian Tumors with Low CCL23 or High CXCL10 Expression Correlate with Improved Patient Survival

**Figure 3:**
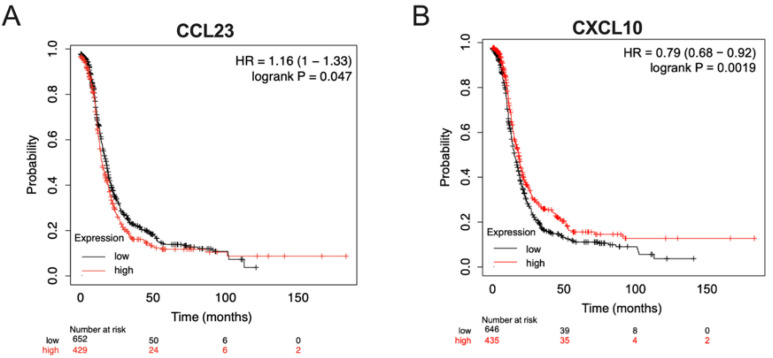
In silico analysis of patient outcomes differs based on tumor expression levels of CCL23 and CXCL10. Kaplan–Meier overall survival analyses were performed on stage III and IV ovarian cancer patients, histology agnostic (*n* = 1081, http://kmplot.com), using datasets hosted in the Cancer Genome Atlas (TCGA). Patients were stratified based on the quantile expression of high (red line) versus low (black line) (**A**) CCL23 mRNA or (**B**) CXCL10 mRNA expression from ovarian tumors. Log-rank and Cox hazards tests were performed for statistical significance.

To better understand the relationships between CCL23 and CXCL10 expression on patient survival, we performed an in silico assessment of patient outcomes based on the tumor expression levels using datasets hosted in the Cancer Genome Atlas (TCGA) database. For CCL23, patients were separated into two groups based on the quantile expression (high versus low). Kaplan–Meier survival analysis was performed on stage III and IV ovarian cancer patients (*n* = 1081, http://kmplot.com). Patients with low levels of CCL23 tumor mRNA were noted to have significantly better outcomes versus patients with high CCL23 concentrations (HR 1.16 [95% CI 1.0–1.33], *p* = 0.047) (Figure 3A).

Similarly, patients were independently stratified by the quantile expression of CXCL10 tumor mRNA. A significant difference was observed between groups (HR 0.79 [95% CI 0.68–0.92], *p* = 0.001). Women with high CXCL10 tumor expression had a better prognosis compared to those with lower CXCL10 expression levels (Figure 3B). No significant differences were observed between groups in patient-associated clinical data (HR 0.54 [95% CI 0.21–1.36], *p* = 0.38).

### 3.4. CCL23 Stimulation Reduces CXCL10 Secretion from Myeloid Cells

**Figure 4:**
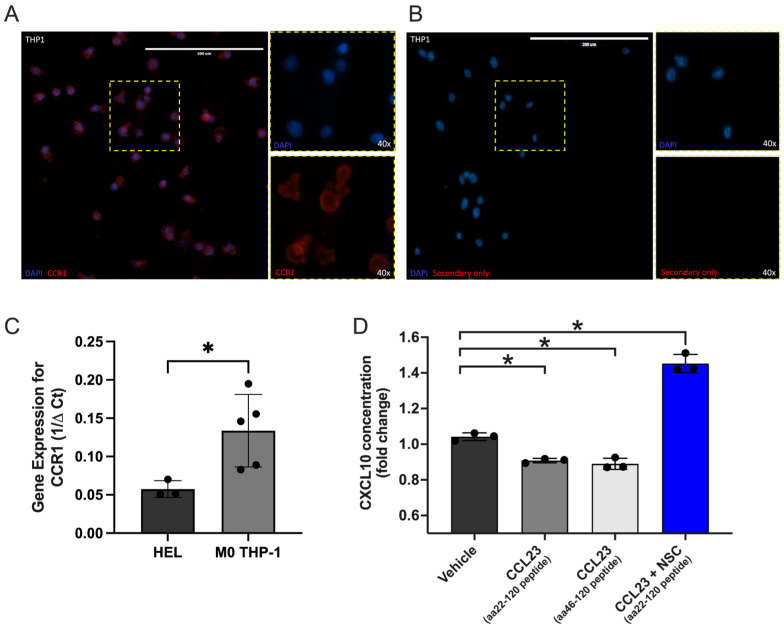
In vitro stimulation of immune cells with CCL23 reduces CXCL10 secretion. (**A**) Immunohistochemical staining of differentiated THP-1 monocytes for the C-C motif chemokine receptor 1 (CCR1; red); nuclei stained with DAPI (blue). Scale bar = 200 μm. (**B**) Secondary antibody only staining control. (**C**) qRT-PCR assessment of gene expression for CCR1 in HEL (*n* = 3) and THP-1 cells (*n* = 3). (**D**) Quantification of C-X-C Motif Chemokine Ligand 10 (CXCL10) cytokine concentrations from cell-free supernatant of treated THP-1 cells represented as fold-change normalized to vehicle control (*n* = 3 per group). Cells were stimulated with either vehicle, CCL23 peptides (aa22-120 and aa46-120; 100 ng/mL, 30 min), or the signal transducer and activator of transcription (STAT-3) inhibitor NSC 74859 (100 μM) followed by CCL23. Data presented as mean ± S.E.M., *n* = 3 separate experiments. Statistical analyses performed using *t*-test or one-way ANOVA with Bonferroni post hoc test for significance; * *p* < 0.05.

CCL23 mediated signaling via the CC chemokine receptor 1 (CCR1) promotes TME formation, tumor cell migration, and colonization of ovarian cancer cells to the omentum. Given the inverse relationship between CCL23 and CXCL10 in human ovarian ascites samples and reduced survival in patients with low tumor CXCL10 expression, we hypothesized that the activation of CCR1 via CCL23 impairs CXCL10 secretion from myeloid cells. We performed in vitro studies using differentiated THP-1 monocytic cells, which express CCR1 at the plasma membrane when differentiated to macrophages (Figure 4A). Gene expression analysis confirmed the presence of CCR1 in THP-1 cells (Figure 4B). HEL cells were used as a control cell line as they have low expression for CCR1.

We next stimulated cells using two different CCL23 peptides (aa22-120 and aa46-120) and measured CXCL10 concentrations from cell-free supernatant by ELISA. Optimal stimulation conditions for CCL23 were determined using cell migration assays that evoked significant increases in cell motility and were previously published by our laboratory [15]. Stimulation of THP-1 cells with either CCL23 peptide significantly decreased CXCL10 secretion versus a vehicle-treated control group (aa22-120 versus control, *p* = 0.019; aa46-120 versus control, *p* = 0.005) (Figure 4C).

STAT3 is known to be activated in many cancers and facilitates cross-regulation of tumor and immune cells [17,18,19]. Additionally, in CD8+ T-cells, STAT3 signaling inhibits CXCL10 production through the reduction in IFNγ expression [20]. Inhibition of STAT3 in this model system was therefore assessed (Figure 4D). Co-incubation of the CCL23 aa22-120 peptide and NSC 74859, a STAT3 inhibitor, restored and significantly increased CXCL10 secretion versus vehicle-treated controls. Together, these data demonstrate that CCL23-mediated reductions in CXCL10 secretion are in part mediated by STAT3 activity.

## 4. Discussion

The current study evaluated the clinical relevance and mechanistic role for the pro-tumorigenic cytokine, CCL23, in human ovarian ascites and cultured macrophages. Our findings show that elevated CCL23 concentrations in ascitic fluid are associated with a reduced overall survival in patients with advanced epithelial ovarian cancer. Our findings also demonstrate that elevated CCL23 levels are associated with reductions in CXCL10, which is partly mediated through STAT3 activity. Thus, CCL23 status may serve as a novel biomarker and indicator of poor patient prognosis, likely as a functional driver of immune suppression within the TME.

CCL23 is the natural ligand for the cognate receptor, CCR1, which promotes tumor-supportive processes such as the recruitment of myeloid cells, endothelial cell migration, angiogenesis, and T-cell exhaustion in ovarian cancer [12,13,14]. The current report builds on prior findings which demonstrated that CCL23 facilitates ovarian cancer metastasis to the omentum and contributes to TME formation [15]. Herein we report that high CCL23 ascites concentrations are associated with significantly lower levels of CXCL10 and PD-1, suggesting that CCL23 plays a central role in remodeling the immune landscape of the TME towards an immunosuppressive phenotype. Our findings further demonstrate that high levels of CCL23 are associated with worsening patient outcomes. Notably, we also observed patient populations containing low and intermediate levels of CCL23 that were not associated with reduced overall survival. Though this finding did not reach statistical significance, a clinically meaningful difference in survival of approximately 3 years was noted, consistent with TCGA-derived survival analyses. It is plausible that a threshold level for a cancer–immune set point exists in the TME in which patients can overcome reduced anti-inflammation and initiate an effective anticancer immune response, shaped by the balance of immune-stimulatory and inhibitory forces, T-cell receptor (TCR) signaling, tumor immunogenicity, and host genetics [21]. Further studies are needed to understand how complex immune interactions shape the TME to promote disease progression and worsening patient outcomes. Additional work is also needed to determine how discrete CCL23 concentrations may generate a TME that is more susceptible to disease progression. Overall, high levels of CCL23 were indicative of reduced patient outcomes, suggesting that CCL23 could be a useful biomarker for assessing immune responsiveness in ovarian cancer patients.

The study also observed a prominent inverse association between CCL23 and CXCL10 levels in ovarian cancer patient samples that correlated with patient survival. CXCL10 is a potent chemoattractant produced in response to IFNγ stimulation that subsequently promotes T-cell trafficking to tumors and is associated with improved survival in multiple cancer types [22,23,24]. In fact, higher CXCL10 levels have been correlated with greater T-cell infiltration in ovarian tumors, while its silencing results in immune exclusion and poor outcomes [24,25]. The modest CXCL10 decrease in vitro likely reflects the limited complexity of the current experimental system, which cannot recapitulate the cellular diversity and signaling dynamics of the tumor microenvironment. In vivo, where disease burden is higher and multiple immune and stromal components interact, these peptides may act synergistically to yield a more robust therapeutic effect. As such, our data support the hypothesis that higher CCL23 expression corresponds with low CXCL10 levels and worsening prognosis as indicated in both patient ascites samples and TCGA datasets.

Lastly, to better understand the mechanism by which CCL23 influences CXCL10 levels, we evaluated CXCL10 levels in vitro using exogenous CCL23 peptides. Our results show that CCL23 stimulation reduces CXCL10 secretion from THP-1 M0 macrophages. Notably, THP1 macrophages represent a simplified version of the greater TME and future studies are needed to further elucidate the nuances of this interaction in primary tumor-associated macrophages. Furthermore, the STAT3 inhibitor NSC 74859 prevents CCL23-induced changes in CXCL10 secretion in a monoculture system. Of note, when a STAT3 inhibitor was applied to this system, there was not only recovery of CXCL10 concentrations but a statistically significant increase in CXCL10. This suggests that there is additionally a CCL23 independent but STAT3-dependent mechanism of CXCL10 regulation in this monoculture system. Further investigation into this mechanism is warranted. Other reports consistently show that STAT3 activation suppresses interferon (IFN)-driven cytokine signaling and is frequently implicated in tumor-mediated immune evasion [17,18,19]. STAT3 interferes with IFNγ production and downstream chemokine signaling, including the suppression of CXCL10 expression and its receptor, CXCR3, which are critical for effective CD8+ T-cell tumor infiltration [20].

Prior studies have also implicated GSK3 in CCL23-mediated T-cell exhaustion [12], and GSK3β is known to interact with STAT3 and STAT5 signaling pathways [26]. These data suggest that CCL23’s effects on the TME are multifaceted and may involve multiple converging regulatory networks. Further studies are needed to confirm the involvement of GSK3 in our system and to further test whether this mechanism is present in patients with CCL23-enriched ascites. Phosphorylated STAT3 is frequently elevated across gynecologic cancers and drives key oncogenic processes such as invasion and metastasis [27,28,29]. As a central transcriptional regulator, activated STAT3 contributes to chemoresistance and tumor progression through both JAK2-dependent and independent pathways, underscoring its broad relevance as a therapeutic target in gynecologic malignancies [27]. Future experiments are needed to examine the phosphorylation status of STAT3 and to understand the cell signaling pathways, which are activated by CCL23 in ovarian and other gynecologic cancers.

Our findings also noted a significant difference in soluble PD-1 with changes to CCL23 concentration (Figure S2). As soluble PD-1 has shown variable prognostic significance across multiple cancers, our observed decrease in soluble PD-1 should be interpreted cautiously and not assumed to reflect improved immune function [30,31,32]. In ovarian cancer, where soluble PD-1 remains less studied than soluble PD-L1, existing data suggest that soluble checkpoint ligands in peritoneal fluid correlate with immune regulation, CA125 levels, and clinical outcomes, underscoring that our soluble PD-1 findings are preliminary and should be further investigated with a dedicated study [33].

One limitation of the current study is the use of an in vitro monoculture system that allowed us to directly assess the effects of CCL23 on THP-1 macrophages. Using this system, we observed a STAT3-dependent reduction in CXCL10 secretion to corroborate CXCL10 levels in patient samples. The THP-1 monocytic cell line was used for this data and future studies are needed to evaluate this data in ascites-derived macrophages or more complex co-culture systems. Future in vivo studies are also required to assess the complex immune interactions between other immune cell populations such as stromal cells. In this case, we would hypothesize that CCL23-mediated immunosuppressive effects would exert a more pronounced impact on anti-tumor immunity, particularly due to its known role in promoting the recruitment and accumulation of exhausted T-cells within the TME [12]. CCL23 has been shown to influence the chemotaxis of specific immune cell populations, including monocytes and T-cells, and its expression may drive a shift toward a dysfunctional immune landscape. Another limitation is the moderate sample size of patients included in the initial ascites analysis. Increasing the number of participants would allow for a more robust survival analysis. This would also allow us to better understand the threshold level of the cancer–immune set point wherein CCL23 in the ascites shifts the balance to a pro-tumor response. This knowledge would allow for possible utilization of CCL23 measurement in ascites as a possible predictor of prognosis.

## 5. Conclusions

As one of the main obstacles for ovarian cancer immunotherapy is the inefficiency of CD8+ T-cell recruitment to tumors, understanding the mechanisms by which we can manipulate the TME is important in understanding the challenges and limitation of immunotherapy in ovarian cancer and also allows us insight into how to circumvent this in the hope of being able to make therapy more efficacious. In order to expand the cohort of patients who respond to therapy, novel therapeutic targets or strategic therapeutic combinations need to be identified.

In summary, our study describes a novel interaction between CCL23 and CXCL10 in the tumor microenvironment, which is also associated clinical findings, indicating reduced overall survival with higher CCL23 and lower CXCL10 levels in human ovarian ascites. This appears to be mediated by STAT3. Such an understanding of the crosstalk between CCL23, STAT3, and CXCL10 provides new insights into future therapeutic targets and improving the responsiveness of ovarian cancers to current immune-oncology therapies.

## Figures and Tables

**Table 1:** Patient characteristics. All values are expressed as the number followed by the percent, except for patient age, which is expressed as median and range in years.

Characteristic	No.	*Percent (%)*
Ovarian Cancer Patients	40	
Age (years)		
Median	59.6	
Range	33–90	
Stage		
IC	2	*5.0*
IIB	1	*2.5*
IIIB	2	*5.0*
IIIC	19	*47.5*
IV	13	*32.5*
Unknown	3	*7.5*
Sample Type		
Primary	33	*82.5*
Recurrent	7	*17.5*
Sensitive	3	*42.9*
Resistant	4	*57.1*
Refractory	0	*0*
Histology		
High Grade Serous	32	*80.0*
Low Grade Serous	1	*2.5*
Endometrioid	1	*2.5*
Clear cell	1	*2.5*
Carcinosarcoma	1	*2.5*
Rhabdomyosarcoma	1	*2.5*
Mesonephric-like adenocarcinoma	1	*2.5*
Adenocarcinoma NOS	1	*2.5*
Mixed	1	*2.5*

## Data Availability

Data made available upon request. Data for patients with ovarian cancer retrieved from TCGA is publicly available at https://portal.gdc.cancer.gov/ (Accessed 1 June 2022).

## Supplementary Materials

The following supporting information can be downloaded at: https://www.mdpi.com/article/10.3390/cancers17243925/s1. Figure S1: Association between CCL23 ascites concentrations and clinical–pathologic characteristics. Figure S2. Soluble PD1 concentrations in ovarian ascites. Table S1. Luminex assay results table. Statistical analyses performed using Bonferroni multiple corrections test for significance; * *p* < 0.05. CCL23 high *n* = 11; intermediate *n* = 12; low *n* = 17.

## Abbreviations

The following abbreviations are used in this manuscript:

ATCC	American Type Culture Collection
BSA	Bovine Serum Albumin
CCR1	CC Chemokine Receptor 1
CCL23	CC Chemokine Ligand 23
CD8+	Cluster of Differentiation 8 Positive
cDNA	Complementary Deoxyribonucleic Acid
CXCL10	C-X-C Motif Chemokine Ligand 10
CXCR3	C-X-C Motif Chemokine Receptor 3
DAPI	4′,6-Diamidino-2-Phenylindole
ELISA	Enzyme-Linked Immunosorbent Assay
FBS	Fetal Bovine Serum
GSK3	Glycogen Synthase Kinase 3
GSK3β	Glycogen Synthase Kinase 3 Beta
HGF	Hepatocyte Growth Factor
HGS	High Grade Serous
HIMC	Human Immune Monitoring Center
HR	Hazard Ratio
ICC	Immunocytochemistry
IFN	Interferon
IRB	Institutional Review Board
KM	Kaplan–Meier
Luminex	Multiplexed Bead-Based Immunoassay System
mOS	Median Overall Survival
mRNA	Messenger Ribonucleic Acid
NACT	Neoadjuvant Chemotherapy
NCCN	National Comprehensive Cancer Network
NSC 74859	STAT3 Pathway Inhibitor (Small Molecule Compound)
PBS	Phosphate-Buffered Saline
PCR	Polymerase Chain Reaction
PD-1	Programmed Cell Death Protein 1
PFA	Paraformaldehyde
PMA	Phorbol 12-Myristate-13-Acetate
qRT-PCR	Quantitative Real-Time Polymerase Chain Reaction
RPM	Revolutions Per Minute
RPMI	Roswell Park Memorial Institute
STAT3	Signal Transducer and Activator of Transcription 3
S.E.M.	Standard Error of the Mean
TCGA	The Cancer Genome Atlas
TCR	T-Cell Receptor
THP-1	Human Monocytic Cell Line
TME	Tumor Microenvironment
18S	18S Ribosomal RNA
