# Supplementary material for: Ovarian Cancer Ascites Enriched for CCL23 Reduces Macrophage-Derived CXCL10 Secretion and Is Associated with Poor Patient Outcomes
Source: Cancers (Basel). 2025 Dec 8;17(24):3925. doi: 10.3390/cancers17243925 (PMC12731045; doi:10.3390/cancers17243925)
Supplement: Supplementary file 1 [file cancers-17-03925-s001.zip › cancers-3990918-supplementary.pdf]

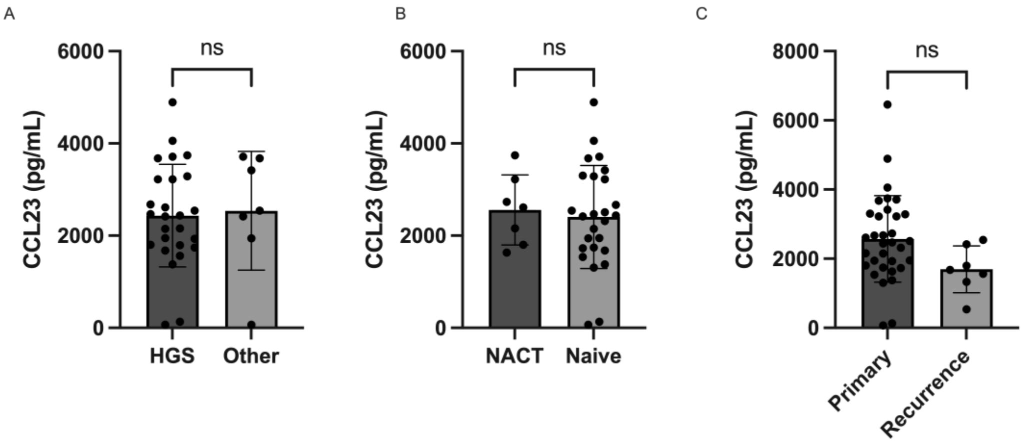

Supplemental Figure S1. Association between CCL23 ascites concentrations and clinical-pathologic characteristics (histology, chemotherapy exposure, primary or recurrent sample; Student's t-test). *HGS – high grade serous; NACT – neoadjuvant chemotherapy.*

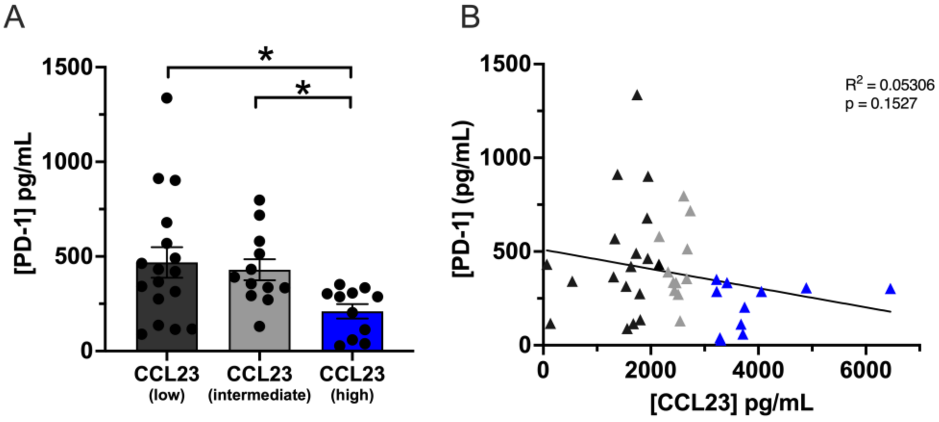

Supplemental Figure S2. Soluble PD1 concentrations in ovarian ascites. (A) Quantification of Programmed cell death protein-1 (PD-1) cytokines from ovarian ascites patient samples containing low (n=17), intermediate (n=12) and high (n=11) CCL23. Data presented as mean ± S.E.M. Statistical analyses were performed using one-way ANOVA with Dunnet's post-hoc test; *p<0.05. (B) Pearson linear regression analysis of PD-1 versus CCL23 concentrations indicated a non-significant negative association between ($F(1,38)=2.13$, p=0.15).

Supplemental Table S1. Luminex assay results table. Statistical analyses performed using Bonferroni multiple corrections test for significance; *p <0.05. CCL23 high n=11; intermediate n=12; low n=17.

Cytokine	Adjusted p-values: Bonferroni's multiple comparisons test		
	CCL23 (high) vs CCL23 (intermediate)	CCL23 (high) vs CCL23 (low)	CCL23 (intermediate) vs CCL23 (low)
CXCL10/IP-10	**0.0195*	**0.0206*	>0.9999
PD-1	**0.0021*	**0.0173*	0.8415
BTLA	**0.0169*	0.714	0.1348

CD27	0.9148	0.3404	>0.9999
CD28	>0.9999	>0.9999	>0.9999
TIM3	>0.9999	0.2807	0.1139
HVEM	0.1884	0.8536	0.8711
CD40	>0.9999	>0.9999	>0.9999
GITR	>0.9999	>0.9999	>0.9999
LAG3	0.9748	0.1155	0.8692
TLR2	>0.9999	>0.9999	>0.9999
GITR	>0.9999	>0.9999	>0.9999
CD152(CTLA4)	>0.9999	>0.9999	>0.9999
CD80	>0.9999	>0.9999	0.553
CD86	>0.9999	0.2923	0.6869
PDL1	0.9851	>0.9999	>0.9999
PDL2	0.5689	>0.9999	0.8156
ICOS	>0.9999	>0.9999	>0.9999
CHEX1	>0.9999	0.9735	0.2067
CHEX2	>0.9999	>0.9999	>0.9999
CHEX3	>0.9999	0.5025	0.4644
CHEX4	>0.9999	>0.9999	0.891
SCD40L	0.2134	>0.9999	0.1619
EGF	>0.9999	>0.9999	0.4015
EOTAXIN/CCL11	>0.9999	0.6298	0.9455
FGF2/FGFB	>0.9999	>0.9999	0.8162
FLT3L	0.0532	0.2648	>0.9999
FRACTALKINE/CX3CL1	0.0788	>0.9999	0.0682
GCSF	>0.9999	0.1048	0.1236
GMCSF	>0.9999	>0.9999	>0.9999
GROA	0.0588	0.3178	0.8863
IFNA2	0.2335	>0.9999	0.7781
IFNG	>0.9999	>0.9999	>0.9999
IL1A	>0.9999	>0.9999	>0.9999
IL1B	0.6022	>0.9999	0.313
IL1RA	>0.9999	>0.9999	>0.9999
IL2	0.8922	>0.9999	>0.9999
IL3	0.8645	0.9865	>0.9999
IL4	>0.9999	>0.9999	>0.9999
IL5	0.7879	>0.9999	0.4022
IL6	>0.9999	>0.9999	0.5984
IL7	>0.9999	>0.9999	>0.9999
IL8/CXCL8	0.2086	>0.9999	0.1046
IL9	0.5044	>0.9999	0.3985
IL10	0.7586	>0.9999	0.1274
IL12P40	>0.9999	0.4814	>0.9999
IL12P70	>0.9999	>0.9999	>0.9999
IL13	>0.9999	>0.9999	>0.9999
IL15	0.378	0.4344	>0.9999
IL17A/CTLA8	0.5679	>0.9999	>0.9999
IL17E/IL-25	>0.9999	0.6995	>0.9999
IL17F	0.3264	0.4779	>0.9999
IL18	>0.9999	>0.9999	0.4808
IL22	>0.9999	>0.9999	0.4808

IL27	>0.9999	>0.9999	>0.9999
MCP1/CCL2	0.5879	0.8574	>0.9999
MCP3/CCL7	>0.9999	>0.9999	>0.9999
MCSF	>0.9999	>0.9999	>0.9999
MDC/CCL22	>0.9999	>0.9999	>0.9999
MIG/CXCL9	0.2853	0.4279	>0.9999
MIP1A/CCL3	0.5317	0.3911	>0.9999
MIP1B/CCL4	>0.9999	>0.9999	>0.9999
PDGFAA	>0.9999	>0.9999	>0.9999
PDGFAB/BB	>0.9999	0.8555	>0.9999
RANTES/CCL5	>0.9999	>0.9999	>0.9999
TGFA	0.4324	>0.9999	0.5732
TNFA	>0.9999	>0.9999	>0.9999
TNFB/LYMPHOTOXINA(LTA)	>0.9999	>0.9999	>0.9999
VEGF	0.4605	0.6356	>0.9999
CD40L	>0.9999	>0.9999	0.8108
4-1BBL/TNFSF9	>0.9999	>0.9999	>0.9999
Arginase-1	>0.9999	0.5979	>0.9999
B7-H2/ICOSL	>0.9999	>0.9999	>0.9999
B7-H3/CD276	0.7316	>0.9999	0.264
5'-NT/CD73	>0.9999	0.4885	>0.9999
B7-H4/VTCN1	>0.9999	>0.9999	>0.9999
APRIL	>0.9999	>0.9999	>0.9999
B7-H5/VISTA	0.9576	>0.9999	>0.9999
CD25/IL-2Ra	0.4846	>0.9999	0.0725
B7-H6	>0.9999	>0.9999	>0.9999
CD137/4-1BB	0.7887	>0.9999	0.5497
Granzyme B	>0.9999	>0.9999	>0.9999
CD226/DNAM-1	>0.9999	0.4211	0.3465
CD30/TNFRSF8	0.2271	>0.9999	0.0818
E-Cadherin	***0.0238**	>0.9999	0.087
FGL1/Hepassocin	>0.9999	0.4508	0.8448
Galectin-1	>0.9999	>0.9999	>0.9999
Galectin-3	0.9809	>0.9999	>0.9999
Granulysin	0.7484	>0.9999	>0.9999
IDO1	0.6972	>0.9999	0.1676
MICA	0.7084	0.6634	>0.9999
MICB	0.1776	0.2093	>0.9999
Nectin-2	0.1776	0.2093	>0.9999
BAFF/BLyS	>0.9999	>0.9999	>0.9999
Nectin-4	>0.9999	>0.9999	>0.9999
OX40/CD134	0.6315	0.9366	>0.9999
PVR/CD155	0.7966	0.0715	0.8389
Siglec-7	>0.9999	>0.9999	>0.9999
Siglec-9	>0.9999	0.4829	0.2058
Perforin	0.9957	0.4182	0.4749
